# Lessons on the COVID-19 pandemic: who are the most affected

**DOI:** 10.1038/s41598-023-36493-7

**Published:** 2023-06-08

**Authors:** Jun Xie, Xiangdan Piao, Shunsuke Managi

**Affiliations:** 1 Urban Institute & Department of Civil Engineering, Kyushu University, 744 Motooka Nishi-Ku, Fukuoka, 819-0395 Japan; 2 Faculty of Humanities and Social Sciences, Iwate University, 3-18-34 Ueda, Morioka, Iwate 020-8550 Japan

**Keywords:** Human behaviour, Health occupations

## Abstract

The COVID-19 pandemic has led to significant changes in work and lifestyle, impacting occupational mental health. This study examines the time and individual heterogeneity in the pandemic's effects on occupational mental health using panel data from job stress checks spanning 2018 to 2021. On average, there was an initial alleviation of high-stress risk in 2020, followed by a deterioration in 2021. Based on the job demand-resource theory, we identify the group of employees most affected by the pandemic. The findings highlight that employees in unfavorable workplace conditions are more likely to experience substantial adverse impacts. Adequate workplace support, including factors like interpersonal relationships, managerial support, job meaning, control, and work-life balance, is crucial for mitigating high-stress risk. Additionally, during the early phase of the pandemic, engaged employees experienced a slight decline in occupational mental health, while those lacking job resources at their worksite faced higher levels of occupational stress in the subsequent year. These findings offer practical suggestions for person-centered coping strategies to mitigate the pandemic's adverse impact.

## Introduction

The outbreak of COVID-19 and long-period epidemic prevention measures worldwide have significantly impacted the economy and society, leading to changes in work and lifestyle. Large numbers of industries must face the challenge of retaining the labor force and transforming the working settings. Consequently, the employees' occupational stress becomes a potential and long-lasting issue when the physical and mental workplace conditions cannot adapt to the dramatically changing society accordingly^1^. Despite efforts to strengthen the public healthcare system and economic recovery, the issue of occupational stress continues to be a challenge as the "new normal" of work and lifestyle becomes gradually established. The lack of social communication and the blurred boundary between work and personal life may contribute to occupational hazards such as burnout and other mental health problems^2^. The changes brought about by COVID-19, and the experience during the pandemic could be a good opportunity to redesign the workplace more sustainably and flexibly to meet the unique needs of individual workers and industries^3^. Notably, employees with high occupational stress may need more supportive workplace resources to reduce the risk of burnout and related mental or physical diseases^4^. A workplace design that cares about high-stress employees will lead to a healthier and more supportive work environment for all employees. Risk analyses associated with the COVID-19 pandemic could help understand what kind of workplace environment will be more resilient to the changing work style and provide evidence for better job design with long-term benefits.

In the early phase of the pandemic, plenty of studies concluded a negative impact of the pandemic on workers' mental health and provided practical suggestions to mitigate the negative impacts^5,6^. However, studies usually focus on the impacts during the early COVID-19 outbreak, and few studies present consequent evidence of the continuing epidemic prevention policies on employee well-being along with economic recovery. On the other hand, many studies have focused on industries on the front line of the pandemic, such as healthcare, education, and the service sector. Employees in these sectors often face increased stress levels due to the nature of their work and the challenges brought about by the pandemic, but it is important to remember that the pandemic has greatly impacted individuals and organizations across all industries. Further investigation into multiple industries, such as manufacturing, retail, finance, technology, etc., could provide a better understanding of the pandemic impacts on employees' stress levels. Furthermore, an in-depth analysis of the individual heterogeneity could reveal the specific challenges and coping mechanisms and lead to specific interventions tailored to different workplace environments.

This study aims to answer research questions about how the pandemic affects occupational mental health in different phases of COVID-19 and what kind of workplace employees are more resilient to the changing environment. Based on large-scale panel data during and after COVID-19, this study explores the impacts of the pandemic on employees' occupational mental health and high-stress risk. Additionally, using the sorted effect method (SEM) allows us to examine the impact of the pandemic on different subgroups distinguished by workplace factors.

## Research questions

The COVID-19 pandemic has caused tremendous loss of lives and led to a psychiatric epidemic brought by sudden and widespread changes, such as lockdowns, quarantine, and social distancing. Epidemic prevention measures are considered effective ways to protect lives, but the dramatically changing society under those policies may potentially impact mental health, especially occupational stress^7^. General populations affected by COVID-19 may have a high burden of mental health problems. However, the pandemic's mental health impacts change across various factors, such as age, gender, education, income, occupation, pre-existing mental health conditions, etc.^8–10^. Similarly, occupational stress has also been affected in various ways, as flexible work arrangements (FWA) have been widely adopted during the pandemic, such as remote working and flexible working hours. From the personal resource allocation perspective, employees have limited personal resources, such as time, energy, and finances, which need to be allocated effectively to meet their own work and life demands^11^. The change in work style and the consequent work-life conflicts are considered the primary stressor during the pandemic. Related studies suggest that FWA is positively related to better physical health, reduced absenteeism, and fewer somatic symptoms^12^ and is also effective in mitigating the adverse impact of work-life conflicts^13^. Notably, these positive effects of FWA are found to vary across the type of flexibility in time and space^12,13^. Though most companies are adopting FWA during the pandemic, it is not uniformly beneficial for occupational mental health. The progress of FWA adoption varies among firms, which may lead to different results in employees' occupational mental health.

Prior studies usually focus on the pandemic impact on occupational stress in the early period of the pandemic^14^, while the consequent impacts of COVID-19 in the following years are seldom studied. Related studies suggest that employee expectations of future working conditions and job design are changing in the post-COVID-19 era^15^. The different stages of the pandemic may lead to different challenges in workplace management but also an opportunity for redesigning working settings that supports employee well-being and productivity^3^. Switching to remote work mostly happened in the early phase of the pandemic^16^, while companies without prior experience with FWA may encounter difficulties in implementing effective flexible work management. Additionally, the shift to FWA may not ensure appropriate workplace supports for employees. In the following years, even though not coercive, epidemiological prevention measures continue impacting mental health^17^. Companies need to reassess their conventional work policies and expectations to adjust effectively to the emerging reality of FWA^15^. Following this view, this study compares the impacts on occupational stress during and after the outbreak of the pandemic to examine the time heterogeneity in the pandemic impacts on occupational mental health.

### Research question 1

Do the impacts of the pandemic on occupational mental health vary across different pandemic phases?

Besides the different phases of the pandemic, another critical factor thought to be changing the pandemic's impacts on occupational mental health is the industry. Plenty of studies examined occupational stress in the sectors that are directly affected by the pandemic, such as healthcare, hospitality, restaurant, police, etc.^5,6,18–23^. Among the prior studies, occupational stress of workers in the healthcare sector has been the most focused issue^6,24–29^. During the pandemic, hospital workers reported increased psychological stress, lower self-rated health status, and worse physical health^30^. Meanwhile, studies also noted that the COVID-19 pandemic had caused psychological distress in healthcare workers and non-healthcare workers without statistically significant differences, given the combined effect of anxiety, depression, PTSD, and occupational stress^31^. For other industries, prior studies also discussed the impact of COVID-19 on hotel employees^32^, restaurant employees^5^, teachers^33^, and police officers^22^. Furthermore, a cross-industrial comparison has also been conducted to show the industrial heterogeneity in the impacts on occupational stress.

However, industry or firm-level studies fail to capture individual disparities and ignore the chance to offer person-centered coping suggestions. At the individual level, working parents have been particularly vulnerable as they have had to balance the daily changes in demands of their jobs with the responsibilities of caring for their children^34^. Wu^35^ also noted gendered disparities in working hour change of remote workers during COVID-19, indicating the individual difference facing work-life balance challenges. From the job demands-resources model perspective, employees' stressors directly come from their workplace conditions, categorized into job demands and resources^36–38^. On the job demand side, physical or emotional demands at work cause exhaustion, job-related anxieties, etc., which detriments job performance. In the worst case, overloaded job demands are related to high stress and burnout^37,39^. On the resource side, physical or psychological supports at the workplace play a vital role in enhancing engagement and mitigating the adverse effects due to the lack of job demands^40^. Individual workplace-level analyses of the pandemic impact could reveal a more specific map about which kinds of workplaces are more resilient during the uncertainty of work^41^. Thus, research question 2 is about whether the impacts of the pandemic on occupational mental health vary across individual workplace factors and, if so, which kind of workplace is more resilient.

### Research question 2

Do the impacts of the pandemic on occupational mental health vary across personal workplace factors?

## Materials and methods

We constructed a four-year panel dataset from 2018 to 2021 from the stress check program, a national occupational health policy launched by the Japanese government and mandatory for all workplaces with 50 or more employees^42,43^. Employees covered by the stress check program must take the stress check survey at least once a year. This four-year tracking survey dataset contains 88,781 unique employees each year and is provided by a third-party company that helps implement the stress check program. The stress check survey asks about employees' occupational stress and 31 workplace factors in job burdens and resources (see Appendix Fig. S1 for correlation between key variables). Female employees stand for 36%, and employees working in foreign companies consist of 5%. The detailed descriptive statistics by year are shown in Appendix Tables S1 and S2. This dataset has the strength of broad coverage of industries such as Manufacturing, Information and communications, Finance and insurance, Wholesale and retail trade, etc., which allow us to have in-depth research about the impacts of the pandemic. The age groups range from 20 to 60 s (see Appendix Table S4).

As shown in Eq. (1), the baseline model aims to investigate high-stress risk and occupational mental health trends from 2018 to 2021 by setting the year 2018 as the reference year and controlling for gender, age group, sector, foreign company dummy, and firm denoted by ${X}_{it}$. ${Outcomes}_{it}$ denotes two dependent variables: high-stress risk and occupational mental health. High-stress risk is a dummy variable indicating whether the employee is of high occupational stress. Occupational mental health measures employees' psychological stress reactions at work in the recent month, covering 18 stress check items in five aspects of vigor, irritability, fatigue, anxiety, and depressed mood. These items are all 4-point Likert Scale. The original score collected from the survey result is recalculated to standard scores ranging from 0 to 100. A higher score means better occupational mental health. High occupational stress in the workplace can, albeit infrequently, lead to a hazardous situation where employees face an increased risk of burnout and related mental or physical health issues^4^. Therefore, we focus on the high-stress risk, which refers to the probability of being a high-stress employee. According to the official guidance of the stress check program, there are two criteria to identify high-stress employees. Criteria 1 depends on the 18 stress check items (see Appendix Table S3), in which an original score higher than 77 is considered high stress. Criteria 2 depends on both the 18 stress check items and 11 workplace factors (see Appendix Table S2: Q1 ~ 17 and Q47 ~ 55). Employees with the original score of 18 stress check items higher than 63 and 11 workplace factors higher than 76 will be identified as high-stress employees. This study focuses on the pandemic impact on occupational stress rather than workplace environments and aims to distinguish the heterogeneity of the impacts across workplace factors. Thus, we choose Criteria 1 to identify high-stress employees.

We use a year dummy to capture the change in the working environment and society during COVID-19. There were four times of emergency announcements in Japan in 2020 and 2021. The first emergency announcement started in April and extended to May 2020. The following three times of emergency announcements are from January to September with short intervals of release and almost covered the stress check survey period in 2021. These prevention policies are considered the most influential factors in changing work and lifestyle, similar to those implemented by other countries, such as social distancing, quarantine, and testing. During the state of emergency, the government has asked non-essential businesses to shorten their service time and asked residents to stay home as much as possible. The factories and companies have been asked to implement flexible or remote working hours if possible. Once the emergency announcement has been lifted, it might be re-imposed depending on the regional pandemic's situation. The shift to remote working, shortened working hours, and reduced capacity in certain businesses have all impacted the economy and daily life. These measures have also increased some employees' isolation and uncertainty about job security. Thus, the year dummy can be used as an effective proxy of the entire workplace and society change. As a robustness check, results based on the emergency announcement period do not significantly change the conclusion.

1 $${Outcomes}_{it}={\beta }_{0}+{\beta }_{1}Yea{r}_{it}+{X}_{it}\beta$$

Then, we used the sorted effects method (SEM) and classification analysis ^44,45^ to estimate the heterogeneity of the pandemic impact on occupational stress. This approach has been widely applied to investigate the partial effects according to individual heterogeneity^46^. Suppose a regression function ${Outcomes}_{it}=g({T}_{it},{W}_{it})$, where ${T}_{it}$ denotes the ordinary variables of year 2019 to 2021 and ${W}_{it}$ denotes the workplace factors and other covariates, including individual characteristics and organizational attributes. The factors of interest are 31 workplace indicators, including 8 job burdens and 23 job resources. The job resources consist of three levels: task level, group level, and worksite level. All the indicators are recalculated into standard scores ranging from 0 to 100. Organizational attributes include foreign company dummies and sectors, and individual characteristics include age group and gender. The missing value of the age group is imputed according to the trend in previous survey data. Then, the partial effect of T on occupational mental health is as Eq. (2). Instead of summarizing a single average partial effect, the SEM reports the entire set of partial effects, sorted in increasing order and indexed by percentiles set from 2 to 98%. We ran the estimations via 200 bootstrap iterations to produce the confidence intervals.

2 $$\Delta \left(x\right)=g\left(yea{r}_{1}, w\right)-g(yea{r}_{0},w)$$

Classification analysis consists of two steps: first, classifying the observational units with partial effects above or below the 10% most and least affected subgroups; second, comparing the averages for covariates W in each group. Statistical inference is obtained via bootstrapping. Since the high stress is a binary response denoted by $High stres{s}_{it}$, logit model is used to estimate the partial effects. The OLS model with interaction items is applied to estimate the partial effects on ${Occupational mental health}_{it}$.

### Ethics approval and consent to participate

Ethical approval for the study was obtained from the Ethics Committee of PEACEMIND Inc (ref. no.: R03-001). The research was performed in accordance with relevant guidelines and regulations. The individual data regarding the stress check survey was provided with informed consent. The data used in this study does not target personal health information, and personal information is non-identifiable.

## Results

Results in Table 1 suggest that occupational mental health was initially improved during the early stages of the COVID-19 pandemic in 2020, but it returned to pre-pandemic levels in 2021. This trend was observed for both high-stress risk and occupational mental health. By investigating a broader range of industries, the negative impact is found in the following years of the pandemic, which could result from the continuing prevention policies and the established new working style without appropriate work design. The initial decrease in stress was likely due to the novelty of the situation by implementing remote work, which is consistent with the positive effect of FWA in prior studies^12^. However, as the pandemic progressed, the blurring of work-life boundaries may lead to new challenges for workplace management^2^. It is possible that impropriate workplace adjustment deteriorated stress levels again. To further explore the detailed evidence on workplace factors, we use SEM to examine the individual heterogeneity in the workplace and identify how subgroups are affected differently by the pandemic.

**Table 1:** Baseline model results.

	Dependent variables	
	High-stress risk	Occupational mental health
The reference year 2018		
Year 2019	0.072*** (0.014)	− 0.269*** (0.037)
Year 2020	− 0.037*** (0.014)	0.132*** (0.037)
Year 2021	0.122*** (0.014)	− 0.270*** (0.037)
Female	0.347*** (0.011)	− 0.866*** (0.030)
Foreign	0.076*** (0.022)	− 0.116* (0.062)
Age	Yes	Yes
Sector	Yes	Yes
Firm-fixed	Yes	Yes
Constant	− 2.754*** (0.128)	54.540*** (0.267)
Observations	475,776	475,776
Log likelihood	− 148,732.00	
Akaike inf. crit	297,516.10	
R^2^		0.034
Adjusted R^2^		0.033

The significant levels are as follows: *p < 0.1; **p < 0.05; ***p < 0.01.

### High-stress risk

**Figure 1:**
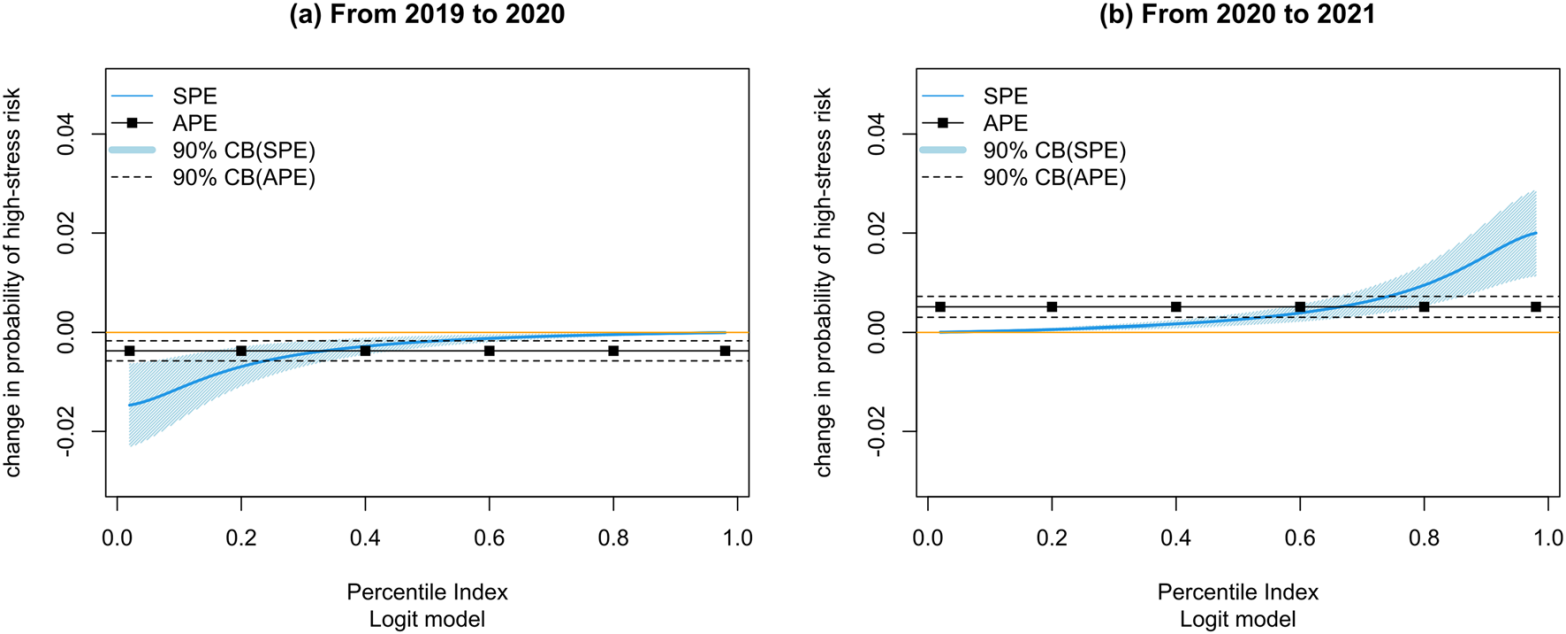
The change in the probability of high-stress risk. This figure shows the results from the sorted effect method: (**a**) the change in high-stress probability from 2019 to 2020 and (**b**) the change in high-stress probability from 2019 to 2020. The black line indicates the average partial effects, and the black dashed line shows a 90% confidence interval. The blue line indicates the sorted partial effects, and the light blue shade offers a 90% confidence interval.

We test the changes in high-stress risk probability from 2019 to 2020 and 2020 to 2021. Figure 1 shows the average partial effects and sorted partial effects of the COVID-19 pandemic on high-stress risk probability. On average, the high-stress risk probability was reduced by 0.37% from 2019 to 2020 and then increased by 0.51% from 2020 to 2021. This result indicates an initial reduction of high-stress risk in the early period of COVID-19 and then a return to the pre-pandemic levels. Furthermore, the results of sorted partial effects show a significant variation in the change of high-stress risk among different population subgroups. Specifically, the maximum reduction in high-stress risk from 2019 to 2020 reached 1.47% in the 2% percentile subgroup, and the maximum increase from 2020 to 2021 reached 2.00% in the 98% percentile subgroup. The yearly average rate of high-stress employees ranges from 9 to 11% in our sample, which means in the worst case, there could be about an 18.2% (2% in 11%) increase in high-stress employees. We further compared the high-stress risk change from 2018 to 2019, before the COVID-19 pandemic. The results suggest an average increase of high-stress risk of about 0.20%, which is weaker than the effects during the pandemic. The partial effects of the more negatively affected subgroup (above 90% percentile) have no significant difference from the average level, indicating considerable changes in high-stress risk due to the pandemic (See Appendix Fig. S2). Additionally, we examine the subsamples before and after the emergency announcement period in 2020 and 2021, respectively, suggesting consistent results (see Appendix Fig. S3). These results show that the impact of the pandemic on high-stress risk is not uniform.

**Figure 2:**
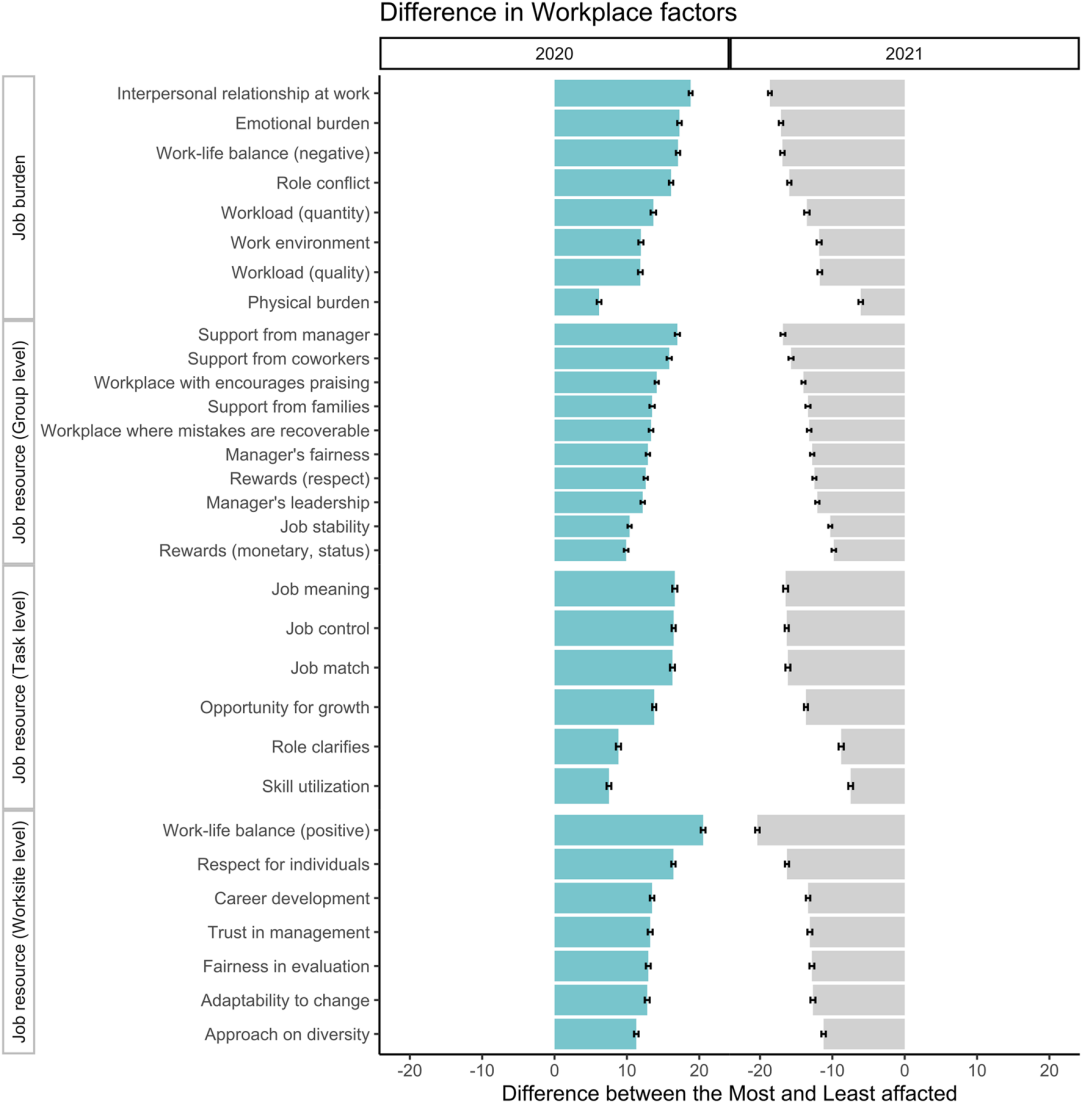
The difference in workplace factors between the unaffected group and the affected group. In 2020, the differences compared the 10% most unaffected subgroup with the 10% most improved subgroup. In 2021, differences compared the 10% most deteriorated subgroup to the 10% most unaffected subgroup. The error bar indicates a 99% confidence interval.

Classification analysis further explored the differences in individual workplace factors for the 10% most and 10% least affected subgroups. Results in Fig. 2 show the differences in workplace factors between the most and least affected subgroups (see details in Appendix Table S5). All the differences are statistically significant, suggesting that high-stress employees are in a very different work environment than those who are not. The subgroup with lower scores in workplace factors experienced both an initial decrease and subsequent increase in high-stress risk. The significant fluctuation at the high-stress levels is risky for high-stress employees, which needs urgent mitigation policies to prevent further detriments. Conversely, employees with better workplace factors are almost unaffected by the pandemic. These results suggest that a better workplace is essential to help employees adapt to changing workstyle during the pandemic.

By exploring the categories based on the job demands-resources theory, we identified the most influential workplace factors: interpersonal relationships at work in the job demands category, support from managers, job meaning, job control, and work-life balance in the three job resource categories. Improving these identified workplace factors is expected to buffer the shock of the pandemic on high-stress risk. For example, by improving interpersonal relationships at work, employees can have a sense of social support which can help them to cope with the stressful situations caused by the pandemic^47^. Employees can access the information, feedback, and guidance they need to manage job demands by providing managers' support. Notably, during the pandemic, job meaning and job control are critical factors that can help employees feel that their work is meaningful and that they have a degree of autonomy in their work^48^. Improving work-life balance can help prevent work-related stress's spillover into employees' personal lives. It is important to note that the effect of the pandemic on stress levels is complex and multifaceted. Improving these factors can help to buffer the shock of the pandemic on high-stress risk, but it may not be enough to mitigate the negative effects of the pandemic completely. As shown in Fig. 2, all 31 workplace factors significantly differ between the most and least affected groups. The factors discussed above have a higher priority in effectively mitigating the negative pandemic impacts than the other factors. In summary, these results answer research question 2 about the individual heterogeneity of the pandemic impacts on high-stress risk, which largely depends on personal workplace factors.

### Occupational mental health

**Figure 3:**
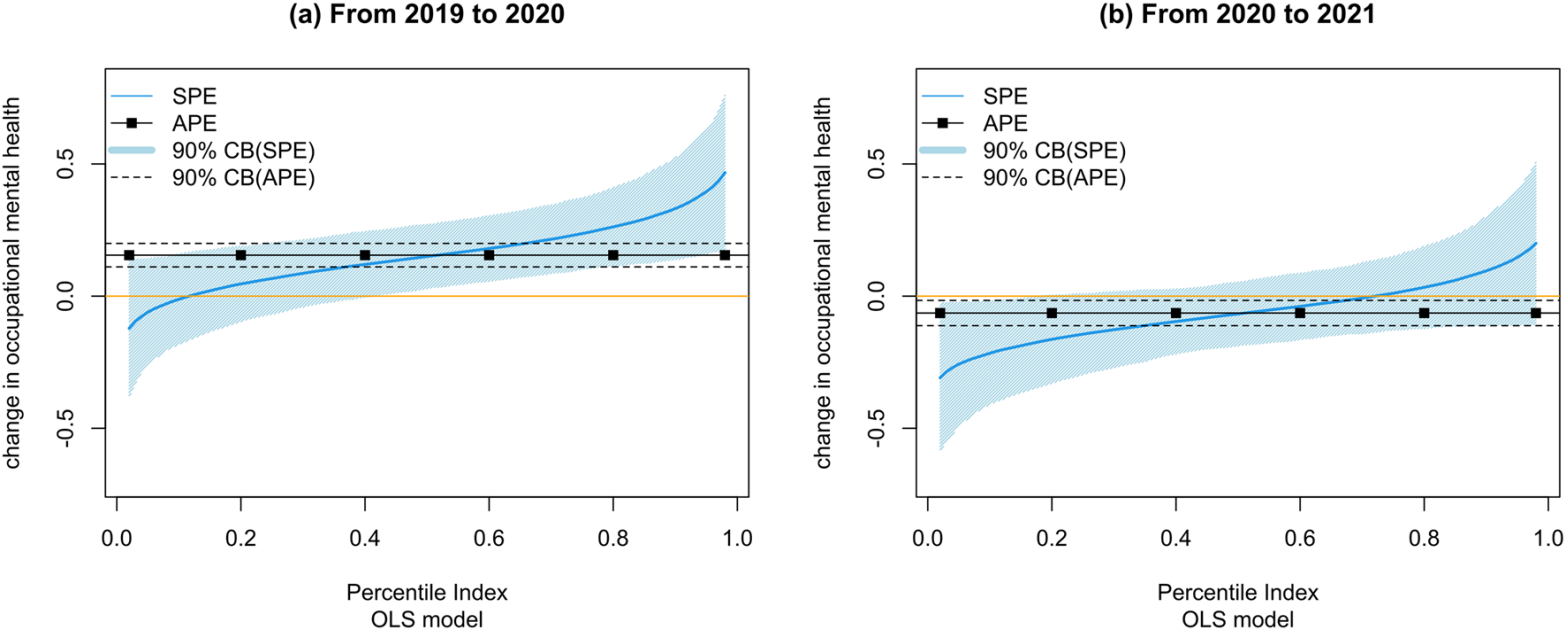
The change in occupational mental health. This figure shows the results from the sorted effect method: (**a**) the change in occupational mental health from 2019 to 2020 and (**b**) the change in occupational mental health from 2019 to 2020. The black line indicates the average partial effects, and the black dashed line shows a 90% confidence interval. The blue line indicates the sorted partial effects, and the light blue shade offers a 90% confidence interval.

Figure 3 shows the change in occupational mental health from 2019 to 2020 and from 2020 to 2021. On average, occupational mental health was improved by 0.15 from 2019 to 2020, while the sorted partial effects range from − 0.12 to 0.47. On the other hand, the average change in occupational mental health was about − 0.06 from 2020 to 2021, while the sorted partial effects range from − 0.31 to 0.2. These trends are consistent with those of high-stress risk, indicating that the pandemic's subsequent impacts may also be more severe than its initial impacts on general employees. However, the heterogeneity of sorted partial effects is not that significant. The impacts on occupational stress are relatively weaker but more widespread across employees. The emergency announcement period studies show consistent results in Appendix Fig. S4.

**Figure 4:**
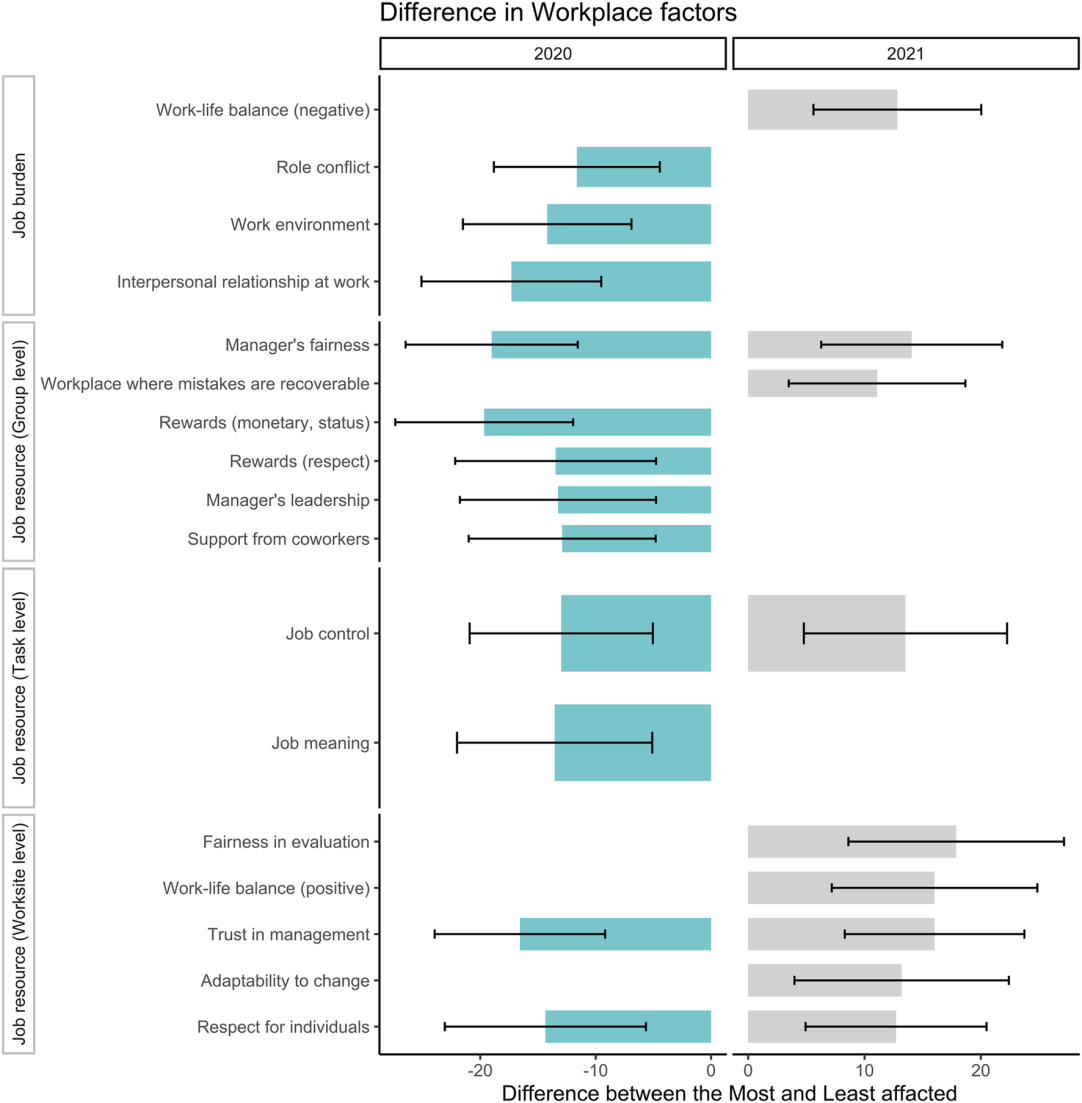
The difference in workplace factors between positively affected group and negatively affected group. This figure only shows the results at the significant level of jointed p-value less than 0.01. In 2020 and 2021, the differences compared the 10% most positively affected subgroup with the 10% most negatively affected subgroup. The error bar indicates a 99% confidence interval.

Still, we can distinguish the 10% most (positively) and 10% least (negatively) affected subgroups. The classification analysis results in Fig. 4 show the difference in workplace factors between the most and least affected subgroups at the significant level of jointed P value less than 0.01 (see detailed results in Appendix Table S6). Interestingly, employees who are most negatively affected by the pandemic in 2020 are those with higher scores of workplace factors, such as interpersonal relationships at work, monetary or status rewards, job meaning, trust in management, etc., which lead to greater work engagement^49^. A possible explanation is that these engaged employees take more responsibility to rethink the strategies to cope with the pandemic, such as new job designs and work styles. These tasks could be a stressor for engaged employees but to a controllable degree without developing into high-stress risks. Quite the opposite, in the early phase of the pandemic, a reduced frequency of being physically present in the workplace provided less engaged employees temporary relief from stressors within the workplace. Meanwhile, employees with fewer workplace supports had a more challenging time adapting to the changed work and lifestyle brought on by the pandemic, and their occupational mental health deteriorated again. Classification analysis results indicate that employees in worse workplace conditions experienced the most negative impacts of the pandemic, especially worksite-level job resources, including fairness in evaluation, trust in management, adaptability to change, and respect for individuals. In the consequent years of the pandemic, managers need to build and sustain trust by demonstrating transparency, empathy, and accountability^50^. Ensuring that evaluations are conducted objectively and based on clear criteria can help to alleviate employee concerns and maintain trust in management. Furthermore, a workplace culture that values recoverability from mistakes can help organizations cope with the uncertainty of work facing new work styles during the pandemic. Finally, work-life balance and job control are always crucial in mitigating the adverse impacts of the pandemic on high-stress risk and occupational mental health.

## Conclusion

The profound change in work and lifestyle brought by the COVID-19 pandemic has had a wide range of impacts on individuals and organizations. During this period, organizations have tried redesigning their working style to maintain business and retain their labor force. The shift to remote work, reduced working hours, and other flexible work arrangements in work practices could all impact employees' occupational mental health. These changes have led to new challenges and stressors for employees, such as increased isolation, difficulty balancing work and home life, and uncertainty about job security. This study provides valuable insights into how organizations can adapt to the changing workstyle during the pandemic and highlights the importance of considering individual workplace heterogeneity when examining the impact of the pandemic on occupational mental health.

By investigating employees in various industries, our findings suggest that the impact of the pandemic on occupational mental health is not universally negative but varies over time and among individuals. On average, employees initially experienced improved occupational mental health, followed by subsequent deterioration, emphasizing the need to adapt to the changes brought about by the pandemic in the subsequent years. Moreover, employees in unfavorable workplace conditions are more significantly affected by the negative impacts of the pandemic compared to those in better workplace conditions. Providing adequate workplace support is expected to mitigate the unfavorable impacts of the pandemic on high-stress risk, especially interpersonal relationships at work, support from managers, job meaning, job control, and work-life balance. Implementing flexible work arrangements (FWA) does not uniformly benefit employees' occupational mental health. Relative workplace supports are expected to mitigate the unfavorable impacts on occupational mental health, including fairness and trust in management, recoverability from mistakes, adaptability to change, and respect for individuals. These findings could provide practical suggestions for managers and policymakers to adopt appropriate and effective person-centered interventions to enhance employees' resilience to the changing work style in a transitioning period.

There are several limitations to consider in our study. First, the investigated sample only covers employees with stable employment states who can participate in the job stress check program but fail to include those who lost jobs during the pandemic. This could lead to a survivor bias since job insecurity due to unemployment leads to different reactions and stressors^51,52^. Although the unemployment rate in Japan did not increase dramatically in the investigated period, from 2.4% in 2019 to 2.8% in 2020 and 2021, we are cautious about the discussion and implications, which are limited to employees with stable jobs. Second, the findings from Japan may not be applicable to other countries due to contextual differences. The conventional working conditions in Japan are considered different from other countries, especially in Western countries. Although the shift to FWA due to the pandemic prevention measures is implemented worldwide, it may have different implications for occupational mental health, which need further investigation. Third, this exploratory study of pandemic impact does not identify specific interventions due to the data availability. Our findings suggest potential avenues for testing targeted interventions related to the pandemic and specific workplace factors that may moderate its impact. Future research can expand the examination of individual heterogeneity within workplace settings to samples from different cultural contexts.

## Supplementary Information

Supplementary Information.

## Data Availability

The stress check survey data that support the findings of this study are available from PEACEMIND Inc., but restrictions apply to the availability of these data, which were used under license for the current study, and so are not publicly available. The stress check survey data are however available from the authors upon reasonable request and with permission of PEACEMIND Inc.
