# Supplementary material for: Lessons on the COVID-19 pandemic: who are the most affected
Source: Sci Rep. 2023 Jun 8;13:9365. doi: 10.1038/s41598-023-36493-7 (PMC10249940; doi:10.1038/s41598-023-36493-7)
Supplement: Supplementary file 1 — Supplementary Information. [file 41598_2023_36493_MOESM1_ESM.pdf]

Lessons on the COVID-19 pandemic: who are the most affected

Jun Xie [a], Xiangdan Piao [a, b], Shunsuke Managi [a*]

[a] Urban Institute & Department of Civil Engineering, Kyushu University, 744 Motooka Nishi-ku Fukuoka, 819-0395, Japan

[b] Faculty of Humanities and Social Science, Iwate University, 3-18-34 Ueda, Morioka, Iwate 020-8550, Japan

Appendix

Table S1 shows the descriptive statistics of the sample for the baseline model. **Table S2** shows the descriptive statistics of stress check items by year for the sorted effect method (SEM) estimation. The stress check survey questionnaire of workplace and occupational mental health is listed in **Tables S2 and S3**, respectively. **Table S4** shows the sample distribution across industries and age groups. **Tables S5 and S6** are the results of classification analyses of high-stress risk and occupational mental health. **Figure S1** shows the correlation between key variables. **Figure S2** illustrates the high-stress probability change from 2018 to 2019. **Figures S3 and S4** show the results of high-stress probability change and occupational mental health change due to the emergency announcement.

Table S1. Basic statistics by year for the baseline model

	**2018**		**2019**		**2020**		**2021**	
	Mean	SD	Mean	SD	Mean	SD	Mean	SD
Health (Psychological stress reaction)	51.04	9.08	50.85	9.12	51.30	8.98	50.89	9.29
High stress (Criteria 1)	0.09	0.29	0.10	0.30	0.09	0.29	0.10	0.30
Female	0.36	0.48	0.36	0.48	0.36	0.48	0.36	0.48
Foreign company	0.05	0.22	0.05	0.22	0.05	0.22	0.05	0.22

Notes: The panel data contains 118,944 observations for each year.

Table S2. Basic statistics by year for the SEM estimation

				2018		2019		2020		2021	
				Mean	SD	Mean	SD	Mean	SD	Mean	SD
**Dependent variables**											
	High stress (Criteria 1)			0.10	0.30	0.11	0.31	0.09	0.29	0.11	0.31
	Health (Psychological stress reaction)	Q18-35: Stress check 18 items (see **Table S2**)		50.68	9.13	50.48	9.18	50.96	9.04	50.55	9.35
**Independent variables**											
Burden	Workload (quantity)	Q1	I have to do an extremely large amount of work.	49.09	8.58	48.97	8.56	49.70	8.52	49.12	8.70
		Q2	I cannot finish my work in time.								
		Q3	I have to work very hard.								
	Workload (quality)	Q4	My job requires a lot of concentration.	48.26	8.14	48.17	8.13	48.35	8.10	48.09	8.15
		Q5	My job requires a high level of knowledge and skills.								
		Q6	During work hours I need to always focus on my job.								
	Physical burden	Q7	My job requires high physical burden.	53.60	8.71	53.70	8.66	54.11	8.58	54.09	8.69
	Interpersonal relationship at work	Q12	There are differences in opinion within my team.	49.37	8.95	49.06	8.86	49.31	8.74	49.20	8.83
		Q13	My team does not get along well with other teams.								
		Q14	There is a friendly atmosphere at my workplace.								
	Work environment	Q15	My work environment is poor in terms of noise, lighting, temperature, and ventilation.	50.33	9.46	50.15	9.40	50.59	9.24	50.89	9.24
	Emotional burden	Q58	My work is emotionally loaded.	49.27	8.67	49.07	8.66	49.26	8.62	48.97	8.69

				2018		2019		2020		2021	
				Mean	SD	Mean	SD	Mean	SD	Mean	SD
	Role conflict	Q59	I receive conflicting demands from two or more people.	47.09	8.91	46.87	8.84	47.12	8.82	47.22	8.84
	Work-life balance (negative)	Q75	I cannot enrich my personal life since I am preoccupied with my work.	49.95	8.70	49.98	8.70	50.36	8.53	49.98	8.72
Task	Job control	Q8	I can work at my own pace.	51.22	8.45	51.35	8.45	51.58	8.38	51.42	8.54
		Q9	I can decide on how and in what order to do my job.								
		Q10	My opinion is reflected in how the job gets done at my workplace.								
	Job match	Q16	I feel that my job suits me.	49.07	9.13	48.93	9.15	48.99	9.11	48.72	9.23
	Skill utilization	Q11	My knowledge and skills are underutilized at work.	48.76	8.71	48.60	8.74	48.60	8.71	48.55	8.70
	Job meaning	Q17	I find my work to be meaningful.	49.68	8.96	49.34	9.00	49.41	8.85	48.99	9.02
	Role clarifies	Q60	I understand my duties and responsibilities.	47.14	9.04	47.10	9.01	47.15	8.95	47.11	8.98
	Opportunity for growth	Q61	I have opportunities to enhance my strength at work.	51.48	7.96	51.31	7.96	51.35	7.85	51.08	7.95
Group		Q47-48-49: How comfortable are you to talk to these people?<br>Q50-51-52: How accountable are these people when you ask for help?<br>Q53-54-55: How much do these people listen to you when you confide your personal problems?									
	Support from manager	Q47	Manager(s)	53.34	9.66	53.27	9.74	53.39	9.73	53.48	9.79
		Q50	Manager(s)								
		Q53	Manager(s)								
	Support from coworkers	Q48	Coworker(s)	51.23	9.62	51.00	9.65	50.99	9.64	50.97	9.74
		Q51	Coworker(s)								
		Q54	Coworker(s)								
	Support from families	Q49	Spouse, family members, and friends	48.53	10.51	48.16	10.66	48.17	10.72	48.10	10.87

				2018		2019		2020		2021	
				Mean	SD	Mean	SD	Mean	SD	Mean	SD
		Q52	Spouse, family members, and friends								
		Q55	Spouse, family members, and friends								
	Rewards (monetary, status)	Q62	My salary and bonus match my work.	54.19	8.39	53.77	8.46	54.10	8.36	53.99	8.46
	Rewards (respect)	Q63	My evaluation from my superior is reasonable.	52.50	8.36	52.34	8.47	52.50	8.38	52.43	8.56
	Job stability	Q64	I feel that there is a possibility to lose my job.	52.36	8.26	52.09	8.34	51.70	8.35	51.90	8.41
	Manager's leadership	Q65	My manager gives me opportunities to enhance my capabilities.	55.22	8.30	55.01	8.25	55.02	8.11	54.91	8.19
	Manager's fairness	Q66	My manager treats me with a sincere attitude.	53.86	8.38	53.66	8.32	53.83	8.22	53.83	8.30
	Workplace with encourages praising	Q67	Efforts made are rewarded at my workplace.	52.26	8.70	51.99	8.65	52.04	8.60	51.95	8.68
	Workplace where mistakes are recoverable	Q68	My workplace gives second chances to recover from mistakes made.	54.89	8.09	54.65	8.10	54.68	8.05	54.51	8.15
Worksite	Trust in management	Q69	Information from management is trustworthy.	51.53	8.96	51.21	8.96	51.26	8.97	51.03	9.10
	Adaptability to change	Q70	Employees' opinions are sought after at times of change in the job or workplace.	51.81	8.84	51.54	8.85	51.74	8.79	51.51	8.88
	Respect for individuals	Q71	Each employees' values are respected.	54.86	9.05	54.71	9.03	54.96	8.94	54.85	9.03
	Fairness in evaluation	Q72	Employee evaluation results are fully explained.	54.84	9.33	54.60	9.40	55.04	9.27	55.17	9.35
	Approach on diversity	Q73	Workers are respected regardless of their labor contracts/ forms of employment (i.e., Full-time employees, contracted employees, part-timers, etc.)	52.27	7.82	52.03	7.84	52.35	7.68	52.35	7.73
	Career development	Q74	Motivation-building or career-enhancing education is conducted at my workplace.	53.83	8.65	53.52	8.65	53.43	8.58	53.48	8.68

			2018		2019		2020		2021	
			Mean	SD	Mean	SD	Mean	SD	Mean	SD
Work-life balance (positive)	Q76	My life is enriched from being energized at work.	51.43	9.31	51.19	9.34	51.45	9.31	51.05	9.39
Female			0.31	0.46	0.31	0.46	0.31	0.46	0.31	0.46
Foreign company			0.06	0.25	0.06	0.25	0.06	0.25	0.06	0.25

Notes: The panel data contains 88,781 observations for each year.

Table S3. Stress check 18 items

**Occupational mental health (Psychological stress reaction)**	**Q18-35: Stress check 18 items**	
	Q18	I have been very active
Vigor	Q19	I have been full of energy
	Q20	I have been lively
	Q21	I have felt angry
Irritability	Q22	I have been inwardly annoyed or aggravated
	Q23	I have felt irritable
	Q24	I have felt extremely tired
Fatigue	Q25	I have felt exhausted
	Q26	I have felt weary or listless
	Q27	I have felt tense
Anxiety	Q28	I have felt worried or insecure
	Q29	I have felt restless
	Q30	I have been depressed
	Q31	I have thought that doing anything was a hassle
	Q32	I have been unable to concentrate
Depressed mood	Q33	I have felt gloomy
	Q34	I have felt sad
	Q35	I have felt dizzy

Table S4. Sample distribution across sectors and age

Sector	Percentage
Construction	7.56%
Education, learning support	0.21%
Electricity, gas, heat supply and water	0.15%
Finance and insurance	15.31%
Industries unable to classify	1.81%
Information and communications	15.53%
Manufacturing	23.88%
Medical, health care and welfare	6.90%
Real estate and goods rental and leasing	2.66%
Scientific research, professional and technical services	1.11%
Services, N.E.C.	8.86%
Transport and postal services	0.88%
Wholesale and retail trade	15.13%
Total	100%
**Age group**	**Percentage**
20~29	13.89%
30~39	24.45%
40~49	33.06%
50~59	23.80%
60~69	4.80%
Total	100%

Notes: The panel data contains 88,781 observations for each year.

Table S5. Results of classification analysis on high stress

	2019 to 2020								2020 to 2021							
	Most	SE	Least	SE	Difference Most - Least	SE	Jointed P-vals	Cat P-vals	Most	SE	Least	SE	Difference Most - Least	SE	Jointed P-vals	Cat P-vals
**Job burden**																
Workload (quantity)	**56.450**	0.099	**42.791**	0.069	**13.659**	0.146	0.004	0.000	**42.873**	0.074	**56.397**	0.098	**-13.524**	0.146	0.000	0.000
Workload (quality)	**54.149**	0.087	**42.291**	0.065	**11.858**	0.126	0.004	0.000	**42.369**	0.062	**54.103**	0.087	**-11.734**	0.126	0.000	0.000
Physical burden	**57.370**	0.070	**51.220**	0.075	**6.151**	0.126	0.004	0.000	**51.266**	0.072	**57.346**	0.071	**-6.080**	0.122	0.000	0.000
Interpersonal relationship at work	**59.082**	0.056	**40.276**	0.072	**18.807**	0.102	0.004	0.000	**40.373**	0.069	**59.009**	0.055	**-18.636**	0.100	0.000	0.000
Work environment	**57.278**	0.068	**45.343**	0.085	**11.935**	0.136	0.004	0.000	**45.407**	0.082	**57.231**	0.066	**-11.824**	0.134	0.000	0.000
Emotional burden	**58.585**	0.067	**41.320**	0.068	**17.264**	0.120	0.004	0.000	**41.418**	0.064	**58.518**	0.067	**-17.100**	0.118	0.000	0.000
Role conflict	**56.171**	0.069	**40.066**	0.064	**16.106**	0.116	0.004	0.000	**40.158**	0.067	**56.109**	0.068	**-15.951**	0.112	0.000	0.000
Work-life balance (negative)	**59.184**	0.070	**42.120**	0.082	**17.065**	0.114	0.004	0.000	**42.214**	0.079	**59.118**	0.069	**-16.904**	0.117	0.000	0.000
**Resource (Task level)**																
Job control	**59.588**	0.064	**43.126**	0.073	**16.461**	0.109	0.004	0.000	**43.216**	0.071	**59.523**	0.063	**-16.307**	0.109	0.000	0.000
Job match	**57.173**	0.060	**40.879**	0.095	**16.294**	0.132	0.004	0.000	**40.961**	0.09	**57.109**	0.059	**-16.148**	0.133	0.000	0.000
Skill utilization	**53.058**	0.080	**45.541**	0.071	**7.517**	0.127	0.004	0.000	**45.561**	0.069	**53.03**	0.079	**-7.468**	0.128	0.000	0.000
Job meaning	**57.397**	0.062	**40.777**	0.094	**16.620**	0.136	0.004	0.000	**40.87**	0.09	**57.332**	0.062	**-16.462**	0.132	0.000	0.000
Role clarifies	**53.825**	0.081	**44.993**	0.082	**8.832**	0.137	0.004	0.000	**45.017**	0.079	**53.792**	0.080	**-8.775**	0.138	0.000	0.000
Opportunity for growth	**58.336**	0.057	**44.560**	0.067	**13.776**	0.108	0.004	0.000	**44.631**	0.067	**58.282**	0.057	**-13.652**	0.105	0.000	0.000

Table S5. Results of classification analysis on high stress (continued)

	2019 to 2020								2020 to 2021							
	Most	SE	Least	SE	Difference Most - Least	SE	Jointed P-vals	Cat P-vals	Most	SE	Least	SE	Difference Most - Least	SE	Jointed P-vals	Cat P-vals
**Resource (Group level)**																
Support from manager	**62.740**	0.081	**45.764**	0.072	**16.976**	0.135	0.004	0.000	**45.842**	0.070	**62.674**	0.081	**-16.831**	0.130	0.000	0.000
Support from coworkers	**59.970**	0.082	**44.119**	0.075	**15.851**	0.136	0.004	0.000	**44.191**	0.075	**59.908**	0.082	**-15.717**	0.134	0.000	0.000
Support from families	**55.756**	0.066	**42.271**	0.104	**13.485**	0.141	0.004	0.000	**42.33**	0.106	**55.703**	0.065	**-13.373**	0.135	0.000	0.000
Rewards (monetary, status)	**59.061**	0.071	**49.168**	0.078	**9.893**	0.122	0.004	0.000	**49.225**	0.077	**59.022**	0.069	**-9.797**	0.121	0.000	0.000
Rewards (respect)	**58.983**	0.063	**46.380**	0.075	**12.603**	0.114	0.004	0.000	**46.454**	0.072	**58.935**	0.064	**-12.481**	0.108	0.000	0.000
Job stability	**57.783**	0.059	**47.413**	0.078	**10.370**	0.109	0.004	0.000	**47.455**	0.075	**57.743**	0.058	**-10.288**	0.105	0.000	0.000
Manager's leadership	**61.417**	0.062	**49.232**	0.079	**12.184**	0.115	0.004	0.000	**49.296**	0.079	**61.369**	0.061	**-12.074**	0.113	0.000	0.000
Manager's fairness	**60.709**	0.056	**47.804**	0.073	**12.904**	0.113	0.004	0.000	**47.869**	0.078	**60.658**	0.056	**-12.79**	0.112	0.000	0.000
Workplace with encourages praising	**59.283**	0.062	**45.161**	0.076	**14.122**	0.109	0.004	0.000	**45.233**	0.077	**59.228**	0.061	**-13.995**	0.107	0.000	0.000
Workplace where mistakes are recoverable	**61.677**	0.054	**48.341**	0.078	**13.336**	0.122	0.004	0.000	**48.418**	0.077	**61.625**	0.055	**-13.207**	0.121	0.000	0.000
**Resource (Worksite level)**																
Trust in management	**58.224**	0.072	**45.002**	0.084	**13.222**	0.131	0.004	0.000	45.062	0.081	58.173	0.072	**-13.111**	0.132	0.000	0.000
Adaptability to change	**58.174**	0.067	**45.364**	0.078	**12.811**	0.132	0.004	0.000	45.439	0.08	58.124	0.067	**-12.686**	0.134	0.000	0.000
Respect for individuals	**63.102**	0.066	**46.674**	0.078	**16.428**	0.115	0.004	0.000	46.767	0.074	63.038	0.065	**-16.272**	0.111	0.000	0.000
Fairness in evaluation	**61.821**	0.080	**48.866**	0.076	**12.955**	0.130	0.004	0.000	48.941	0.076	61.771	0.079	**-12.83**	0.131	0.000	0.000
Approach on diversity	**58.425**	0.062	**47.126**	0.079	**11.298**	0.122	0.004	0.000	47.173	0.078	58.381	0.063	**-11.208**	0.120	0.000	0.000
Career development	**60.504**	0.066	**47.022**	0.078	**13.482**	0.118	0.004	0.000	47.095	0.078	60.452	0.065	**-13.357**	0.118	0.000	0.000
Work-life balance (positive)	**61.986**	0.076	**41.452**	0.064	**20.534**	0.127	0.004	0.000	41.545	0.063	61.907	0.076	**-20.361**	0.120	0.000	0.000

Table S6. Results of classification analysis on occupational mental health

	2019 to 2020								2020 to 2021							
	Most	SE	Least	SE	Difference	SE	Jointed P-vals	Cat P-vals	Most	SE	Least	SE	Difference	SE	Jointed P-vals	Cat P-vals
**Job burden**																
Workload (quantity)	**47.463**	1.500	**48.542**	1.778	**-1.079**	3.256	1.000	0.370	**52.114**	1.685	**44.872**	1.530	**7.243**	3.056	0.800	0.009
Workload (quality)	**44.951**	1.344	**49.536**	1.776	**-4.586**	3.162	1.000	0.073	**51.224**	1.586	**43.960**	1.407	**7.264**	2.916	0.700	0.006
Physical burden	**49.708**	1.678	**57.172**	1.562	**-7.464**	3.119	0.785	0.008	**57.258**	1.773	**53.331**	1.561	**3.928**	3.308	1.000	0.118
Interpersonal relationship at work	**39.023**	1.630	**56.330**	1.480	**-17.307**	3.024	0.000	0.000	**50.698**	1.570	**48.064**	1.741	**2.634**	3.313	1.000	0.213
Work environment	**42.575**	1.592	**56.776**	1.435	**-14.201**	2.835	0.000	0.000	**53.984**	1.533	**48.812**	1.660	**5.172**	3.174	1.000	0.052
Emotional burden	**45.627**	1.588	**50.993**	1.817	**-5.366**	3.364	0.995	0.055	**53.406**	1.419	**44.566**	1.453	**8.840**	2.830	0.360	0.001
Role conflict	**40.098**	1.316	**51.731**	1.521	**-11.634**	2.791	0.055	0.000	**50.939**	1.725	**42.455**	1.666	**8.484**	3.463	0.735	0.007
Work-life balance (negative)	**48.686**	1.447	**50.772**	1.622	**-2.086**	3.114	1.000	0.251	**55.432**	1.376	**42.607**	1.483	**12.825**	2.800	0.010	0.000
**Resource (Task level)**																
Job control	**43.954**	1.646	**56.937**	1.420	**-12.983**	3.082	0.045	0.000	**57.778**	1.532	**44.258**	2.047	**13.520**	3.392	0.055	0.000
Job match	**43.204**	1.825	**53.547**	1.680	**-10.343**	3.220	0.320	0.001	**51.745**	1.790	**46.217**	1.852	**5.528**	3.674	1.000	0.066
Skill utilization	**48.147**	1.463	**48.245**	1.687	**-0.098**	3.000	1.000	0.487	**46.689**	1.636	**51.550**	1.274	**-4.861**	2.884	1.000	0.046
Job meaning	**41.281**	1.872	**54.837**	1.461	**-13.556**	3.284	0.055	0.000	**53.763**	1.650	**44.260**	1.922	**9.503**	3.456	0.545	0.003
Role clarifies	**47.380**	1.621	**49.152**	1.719	**-1.772**	3.410	1.000	0.302	**48.411**	1.713	**48.585**	1.790	**-0.173**	3.555	1.000	0.481
Opportunity for growth	**45.415**	1.607	**55.660**	1.508	**-10.244**	3.173	0.310	0.001	**52.617**	1.472	**50.745**	1.440	**1.872**	2.956	1.000	0.263

Table S6. Results of classification analysis on occupational mental health (continued)

	2019 to 2020								2020 to 2021							
	Most	SE	Least	SE	Difference	SE	Jointed P-vals	Cat P-vals	Most	SE	Least	SE	Difference	SE	Jointed P-vals	Cat P-vals
**Resource (Group level)**																
Support from manager	**45.132**	1.920	**59.430**	1.936	**-14.298**	3.892	0.160	0.000	**58.675**	1.712	**48.692**	1.798	**9.982**	3.467	0.485	0.002
Support from coworkers	**44.392**	1.676	**57.295**	1.790	**-12.903**	3.145	0.060	0.000	**54.038**	1.739	**49.389**	1.723	**4.649**	3.438	1.000	0.088
Support from families	**46.850**	1.703	**50.163**	1.821	**-3.313**	3.490	1.000	0.171	**49.494**	1.875	**48.250**	1.655	**1.244**	3.536	1.000	0.363
Rewards (monetary, status)	**42.676**	1.698	**62.335**	1.317	**-19.660**	2.992	0.000	0.000	**59.371**	1.424	**48.373**	1.829	**10.998**	3.038	0.105	0.000
Rewards (respect)	**44.437**	1.765	**57.907**	1.522	**-13.470**	3.380	0.080	0.000	**57.435**	1.496	**47.396**	1.747	**10.039**	3.246	0.370	0.001
Job stability	**48.475**	1.628	**53.716**	1.598	**-5.241**	3.106	0.995	0.046	**53.745**	1.464	**50.098**	1.387	**3.647**	2.826	1.000	0.098
Manager's leadership	**46.867**	1.720	**60.135**	1.485	**-13.267**	3.299	0.080	0.000	**59.731**	1.461	**50.496**	1.694	**9.235**	3.214	0.485	0.002
Manager's fairness	**42.436**	1.664	**61.449**	1.130	**-19.013**	2.896	0.000	0.000	**60.614**	1.253	**46.555**	1.794	**14.059**	3.021	0.005	0.000
Workplace with encourages praising	**47.043**	1.666	**54.447**	1.521	**-7.404**	3.221	0.830	0.011	**56.410**	1.296	**48.300**	1.318	**8.110**	2.631	0.380	0.001
Workplace where mistakes are recoverable	**50.841**	1.434	**56.518**	1.428	**-5.677**	2.536	0.865	0.013	**60.102**	1.293	**49.022**	1.692	**11.080**	2.948	0.085	0.000
**Resource (Worksite level)**																
Trust in management	**41.226**	1.608	**57.794**	1.345	**-16.568**	2.868	0.000	0.000	**58.945**	1.350	**42.920**	1.760	**16.025**	2.998	0.000	0.000
Adaptability to change	**46.149**	1.793	**53.849**	1.812	**-7.700**	3.601	0.925	0.016	**58.173**	1.669	**44.978**	1.907	**13.195**	3.579	0.100	0.000
Respect for individuals	**45.892**	1.878	**60.253**	1.766	**-14.361**	3.384	0.040	0.000	**61.475**	1.511	**48.763**	1.709	**12.713**	3.020	0.015	0.000
Fairness in evaluation	**47.680**	1.561	**59.436**	1.783	**-11.755**	3.262	0.170	0.000	**63.985**	1.600	**46.104**	2.015	**17.881**	3.598	0.005	0.000
Approach on diversity	**46.223**	1.982	**56.276**	1.592	**-10.053**	3.530	0.505	0.002	**57.232**	1.206	**48.160**	1.734	**9.072**	3.049	0.425	0.001
Career development	**47.191**	1.770	**56.587**	1.737	**-9.396**	3.491	0.610	0.004	**58.979**	1.382	**48.628**	1.549	**10.350**	2.952	0.175	0.000
Work-life balance (positive)	**44.544**	1.634	**54.940**	1.773	**-10.397**	3.263	0.325	0.001	**59.014**	1.892	**42.989**	1.524	**16.025**	3.430	0.005	0.000

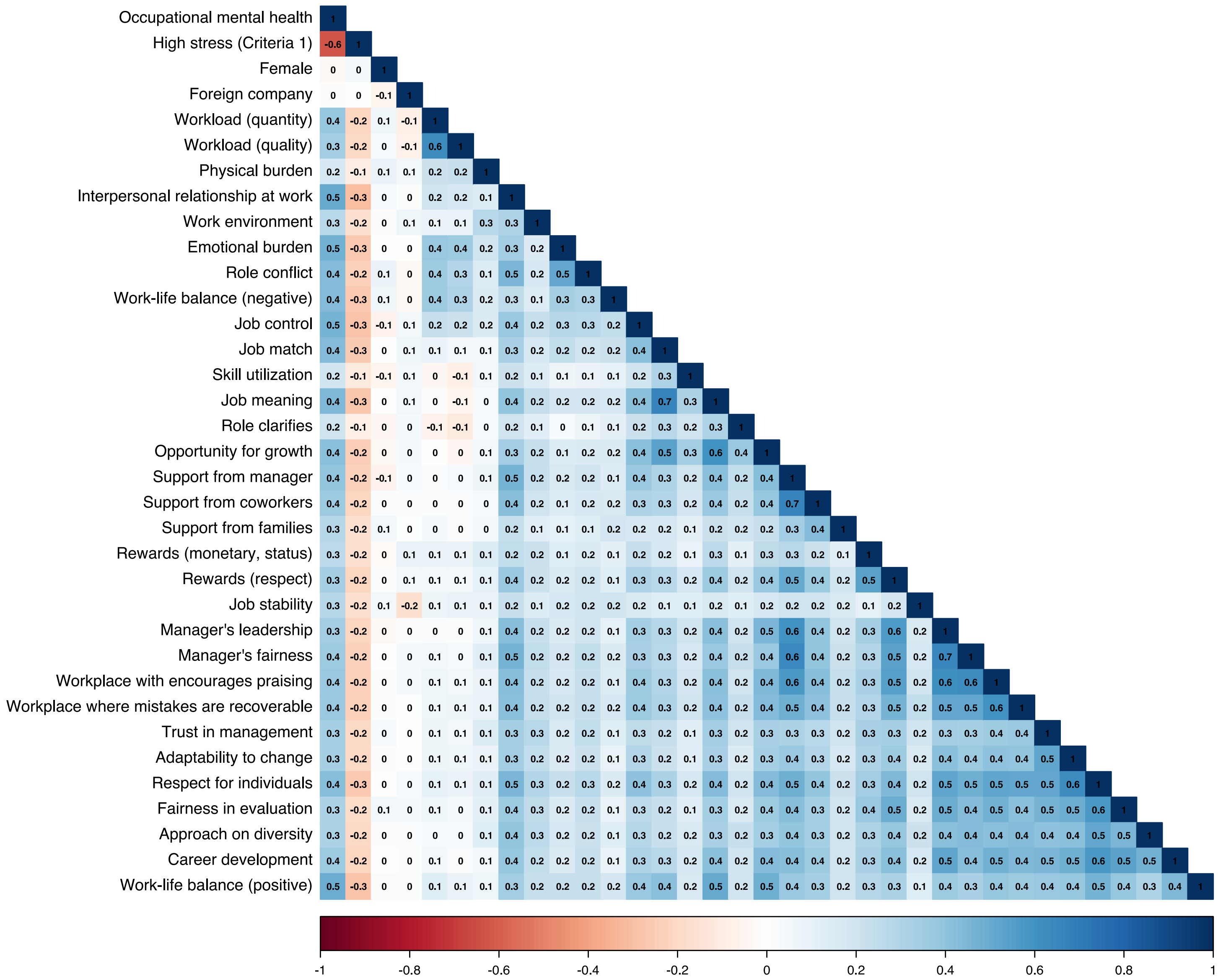

Figure S1. Correlation of key variables

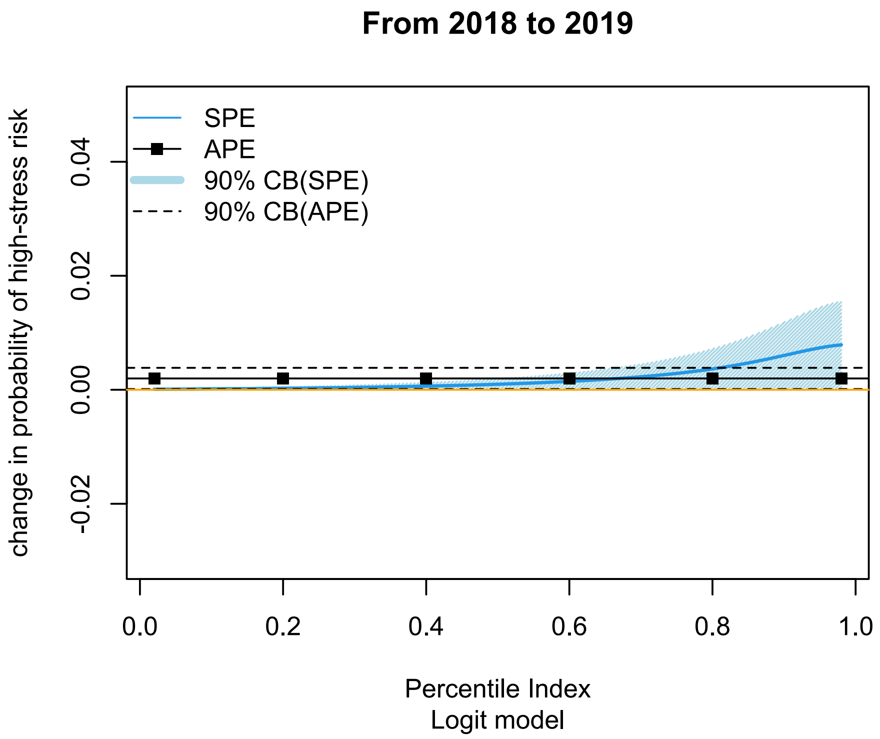

Figure S2. The change in the probability of high-stress risk

Notes: This figure shows the results from the sorted effect method, indicating the change in the probability of high-stress risk from 2018 to 2019. The black line indicates the average partial effects, and the black dashed line shows a 90% confidence interval. The blue line indicates the sorted partial effects, and the light blue shade offers a 90% confidence interval.

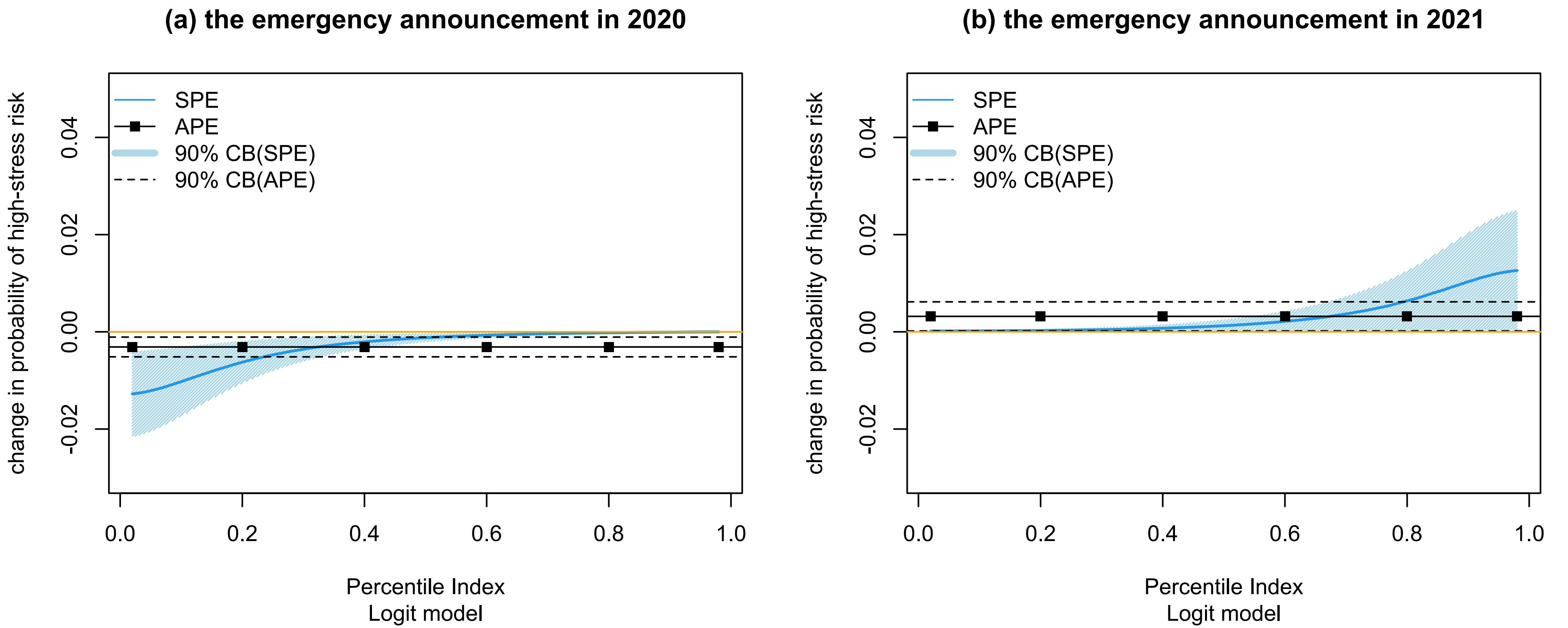

Figure S3. The change in the probability of high-stress risk due to the emergency announcement

Notes: The investigated samples contain (a) 81,323 observations before and after the first emergency announcement in 2020 and (b) 34,628 observations before and after the continuing emergency announcements in 2021. The black line indicates the average partial effects, and the black dashed line shows a 90% confidence interval. The blue line indicates the sorted partial effects, and the light blue shade offers a 90% confidence interval. As shown in panel (a), the result of the emergency announcement in 2020 is consistent with the results using a year dummy in Figure 1 (a). The result in panel (b) shows similar trends as Figure 1 (b) but is not significant since most observations are dropped out due to the long-lasting emergency announcement period in 2021.

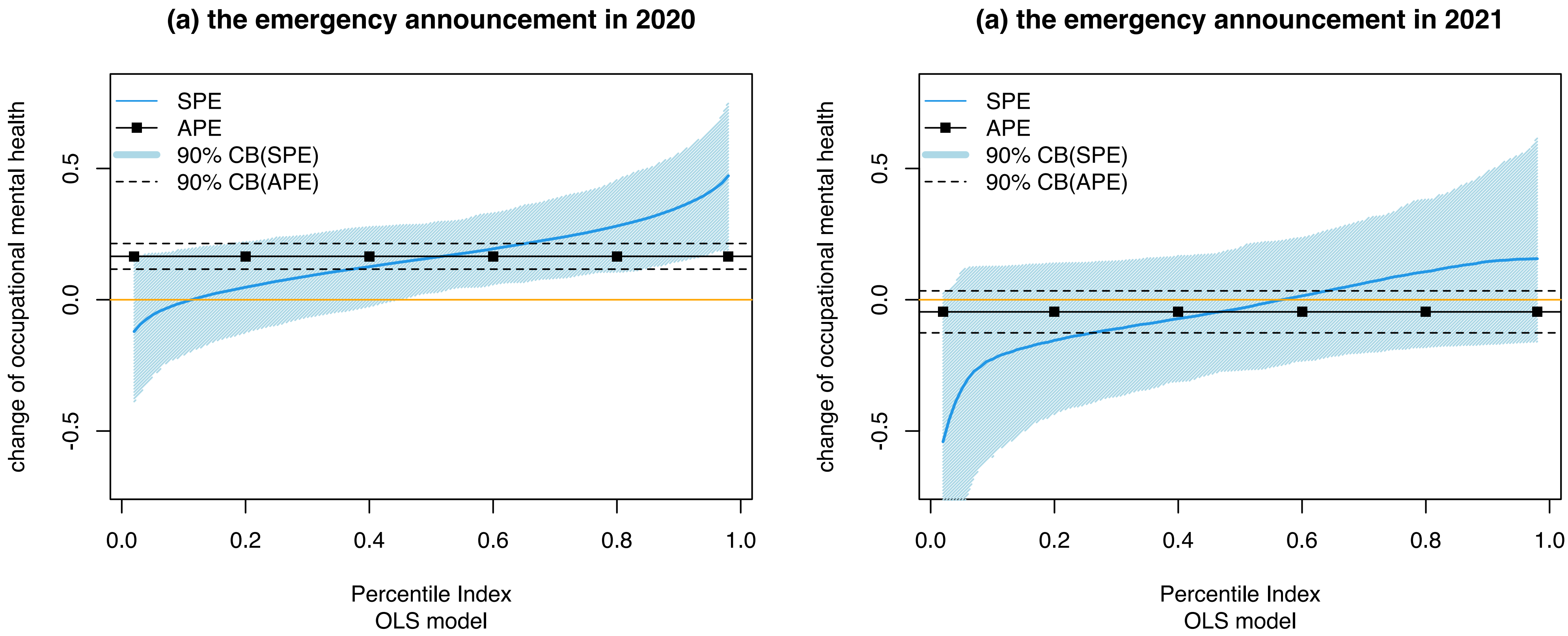

Figure S4. The change in occupational mental health due to the emergency announcement

Notes: The investigated samples contain (a) 81,323 observations during the first emergency announcement in 2020 and (b) 34,628 observations during the emergency announcement in 2021. The black line indicates the average partial effects, and the black dashed line shows a 90% confidence interval. The blue line indicates the sorted partial effects, and the light blue shade offers a 90% confidence interval. As shown in panel (a), the result of the emergency announcement in 2020 is similar to the results using a year dummy in Figure 3 (a), but at a lower significance level. The result in panel (b) is not significant, but the trend is consistent with the results in Figure 3 (b).
